# Lipidomic Signatures of Nonhuman Primates with Radiation-Induced Hematopoietic Syndrome

**DOI:** 10.1038/s41598-017-10299-w

**Published:** 2017-08-29

**Authors:** Evan L. Pannkuk, Evagelia C. Laiakis, Vijay K. Singh, Albert J. Fornace

**Affiliations:** 1 Tumor Biology Program, Lombardi Comprehensive Cancer Center, Georgetown University, Washington, D.C., 20057 USA; 2 Department of Biochemistry and Molecular & Cellular Biology, Georgetown University Medical Center, Washington, D.C., 20057 USA; 3 Department of Pharmacology and Molecular Therapeutics, F. Edward Hébert School of Medicine, Bethesda, USA; 4 Armed Forces Radiobiology Research Institute, Uniformed Services University of the Health Sciences, Bethesda, MD 20814 USA; 5 Deparment of Oncology Georgetown University, Washington, D.C., 20057 USA

**Keywords:** Lipidomics, Systems analysis

## Abstract

Concern over potential exposures of ionizing radiation (IR) to large populations has emphasized the need for rapid and reliable methods of biodosimetry to determine absorbed dose and required triage. Lipidomics has emerged as a powerful technique for large-scale lipid identification and quantification. Indirect effects from IR exposure generate reactive oxygen species (ROS) through water hydrolysis and may subsequently damage cellular lipids. Thus, rapid identification of specific affected lipid molecules represents possible targets for biodosimetry. The current study addresses temporal changes in the serum lipidome from 4 h to 28 d in nonhuman primates (NHPs) with radiation-induced hematopoietic syndrome (6.5 Gy exposure, LD_50/60_). Statistical analyses revealed a highly dynamic temporal response in the serum lipidome after IR exposure. Marked lipidomic perturbations occurred within 24 h post-irradiation along with increases in cytokine levels and C-reactive protein. Decreases were observed in di- and triacylglycerides, sphingomyelins (SMs), lysophosphatidylcholines (LysoPCs), and esterified sterols. Conversely, free fatty acids and monoacylglycerides significantly increased. Decreased levels of SMs and increased levels of LysoPCs may be important markers for biodosimetry ~2 d–3 d post-irradiation. The biphasic and dynamic response to the serum lipidome post-irradiation emphasize the importance of determining the temporal long-term response of possible radiation markers.

## Introduction

Risks of radiation exposure range from nuclear energy accidents to potential terrorism and workplace industrial hazards. Biodosimetry methods are required to aid in the recovery from a potential mass exposure to radiation by determining absorbed dose, assigning triage, and determining useful countermeasures. Much effort has been put into utilizing metabolomics (the collective analysis of molecules <1 kDa) as a way to quantitatively measure markers from radiation exposure from easily accessible biofluids such as urine, serum, and saliva among others^1, 2^. Lipidomics can be considered a branch of metabolomics that aims to catalogue changes in lipid species during injury, disease states, exposure to xenobiotic compounds, or changes in nutritional status^3, 4^. A well-established effect of radiation exposure is the generation of reactive oxygen species (ROS) capable of hydrogen abstraction from biomolecules, such as lipids and proteins, thus causing damage. In addition to IR-induced ROS, cellular responses to IR are known to include proinflammatory-like signaling with ROS further generation^5, 6^. Lipids are prone to peroxidation due to the low carbon-hydrogen bond energies of allylic hydrogen atoms (methylene group hydrogen next to double bonds)^7^. Glycerolipids (GLs) (e.g. triacylglycerides [TGs], diacylglycerides [DGs], monoacylglycerides [MGs]), glycerophospholipids (GPs) (e.g. phosphatidylcholines [PCs], phosphatidylethanolamines [PEs]), cholesteryl esters (CEs), free fatty acids (FFAs), and sphingomyelins (SMs) consist of carbon chains in free acid form or as bound acyl moieties. The acyl moieties can contain no double bonds (saturated), a single double bond (monounsaturated), or multiple double bonds (polyunsaturated). Hydrogen abstraction from these double bonds leads to formation of intermediate peroxidation products that can propagate radical damage to other biomolecules, creating a complex mixture of alkane, alkene, aldehyde, and conjugated diene side products, among others. Due to the high reactivity of lipids to ROS damage, they may be useful in determining absorbed radiation dose for biodosimetry and determining delayed effects of acute radiation exposure (DEARE).

Challenges in lipidomics include the varied and heterogeneous nature of broad lipid classes. The one commonality of lipids is that they are hydrophobic or amphipathic molecules. The result of this broad definition is eight classes with diverse structures ranging from amphipathic ring sterols (e.g., cholesterol), carbohydrate linked saccharolipids, to relatively simple FFAs^8, 9^. While lipid analysis has been performed for decades, many traditional analytical techniques lacked high sensitivity (e.g., thin layer chromatography)^10^ or the ability to simultaneously determine multiple individual lipid species (e.g., fatty acid methyl ester analysis by gas chromatography [GC] mass spectrometry [MS]). With the advent of more sophisticated separation techniques capable of higher resolution and more sensitive detectors, such as ultra performance liquid chromatography (UPLC) coupled with electrospray ionization (ESI) and quadrupole time-of-flight (QTOF) MS, separation and detection of hundreds or thousands can be quickly determined^11^. With the addition of ion mobility spectroscopy and data-independent acquisition, these instruments allow for separation of lipid isomers, collecting *m/z* values of parent compounds with high mass accuracy, and simultaneous MS/MS acquisition in a single analysis^12^.

Previous studies have reported changes in NHP sera lipid levels at 7 d post-irradiation^13, 14^. A global lipidomic analysis reported general decreases in lipid levels but high increases at 10 Gy in lipids with 20:4 (arachidonic acid) and 22:6 (docosahexaenoic acid) acyl moieties, which are important precursors in lipoxygenase (LOX) and cyclooxygenase (COX) mediated inflammation^13^. A subsequent targeted approach confirmed this pattern in PCs and ePCs, where PC (38:6), ePC (40:3) and (40:5) increased at 10 Gy^14^. While these studies indicate clear changes in lipid levels after IR exposure, their scope is limited due to the analysis of a single time point (7 d post-irradiation). In the current study, we performed global lipidomics on NHP serum at time points spanning 4 h to 28 d in order to understand temporal patters in lipid molecular abundance after 6.5 Gy irradiation. While lipid levels are biphasic and highly dynamic after IR exposure, we found high perturbations to the NHP lipidome within the first 24 h post-irradiation. Decreases were observed in DGs, TGs, SMs, and CEs, with minor decreases in LysoPCs within 24 h. Increases were observed in FFAs and MGs, with slight increases in PCs and PEs. Multivariate analysis clearly separates NHPs post-irradiation 3 d–8 d, which are important time points for biodosimetry. Decreased levels of SMs and increased levels of LysoPCs may be important markers ~2 d–3 d post-irradiation. Most lipids returned to pre-irradiation levels after ~8 d, however, higher variability of the serum lipidome at 21 d–28 d may be influenced by bacterial lipids present in the circulation. The biphasic response exhibited by these molecules highlights the importance in timing for sample collection if used for biodosimetry after irradiation.

## Results

### Multivariate Analysis

**Figure 1:**
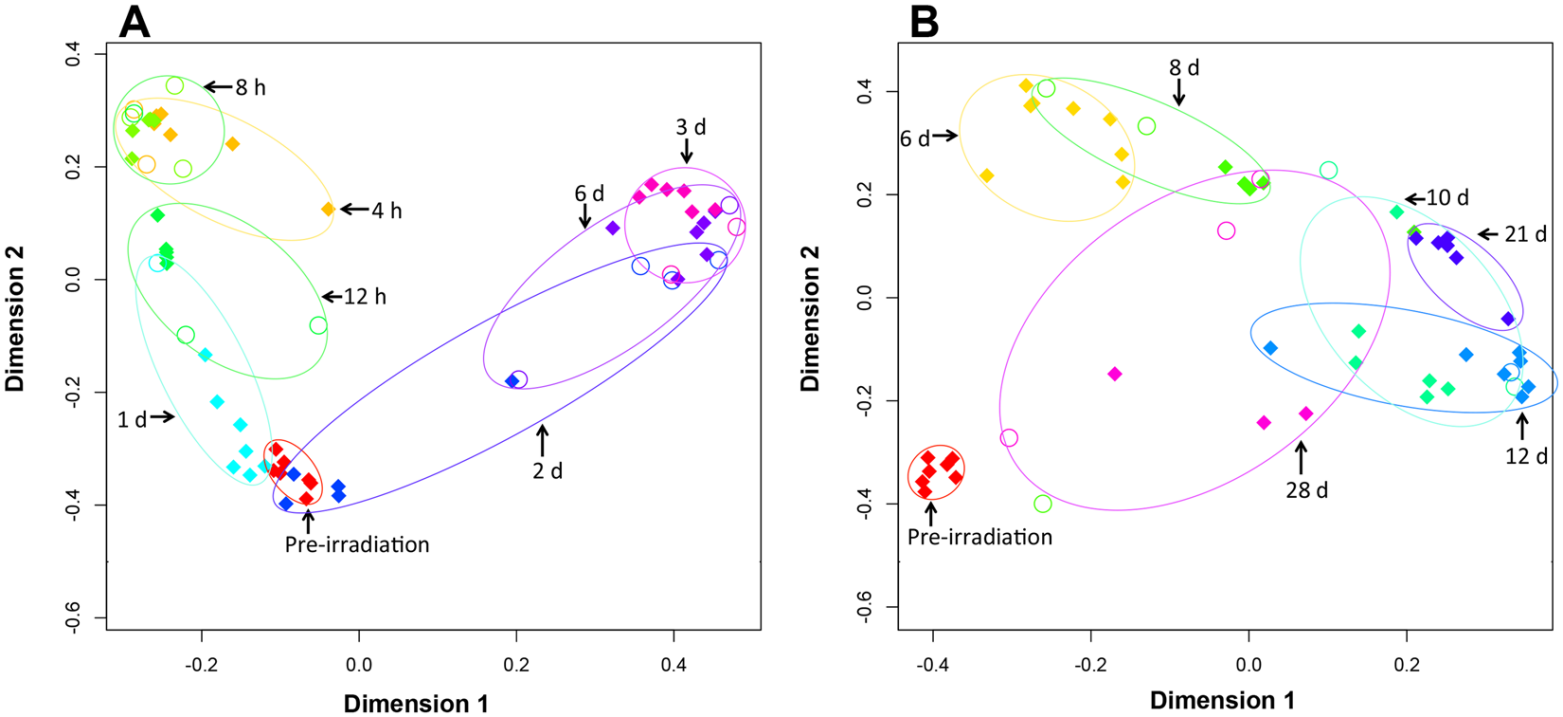
MDS plots generated by the learning algorithm RF comparing lipidomic NHP serum biosignatures (ESI^+^) after exposure to 6.5 Gy γ-radiation. (**A**) Illustrates the first week post-irradiation. Greatest separation occurs along dimension 2 during the initial 24 h and along dimension 1 to 6 d. (**B**) Illustrates 6 d to 28 d, where separation along dimension 1 increases up to 21 d. At 28 d little grouping is observed, which may correspond to increasing bacteria in the circulation.

**Figure 2:**
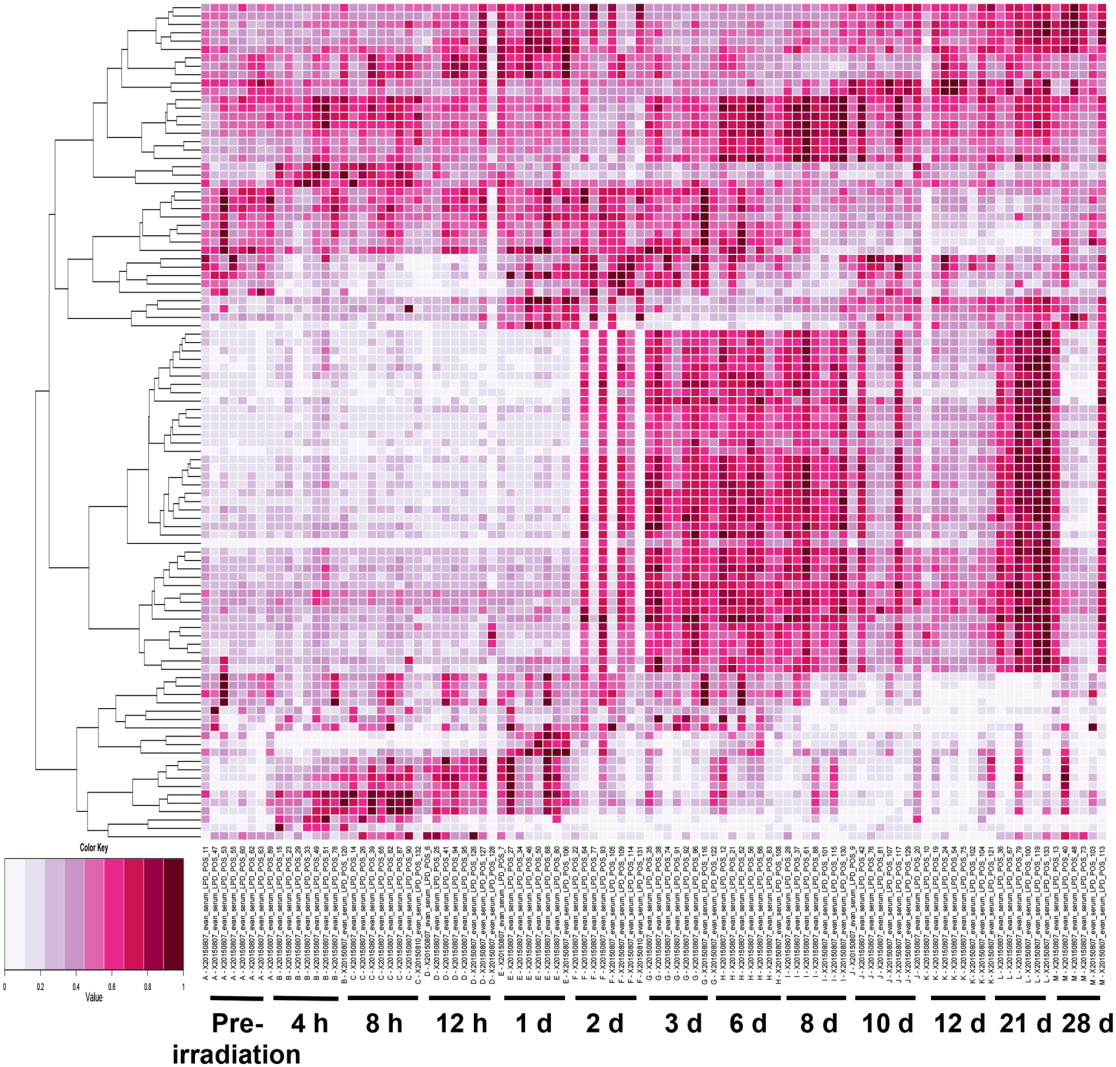
Heatmap generated by the learning algorithm RF comparing lipidomic NHP serum biosignatures (ESI^+^) after exposure to 6.5 Gy γ-radiation. Some ion intensities increase for time point up to 1 d with different shifts in lipidomic signatures from 2 d–8 d and 21 d. *Two NHPs were euthanized before the 21 d time point.

**Figure 3:**
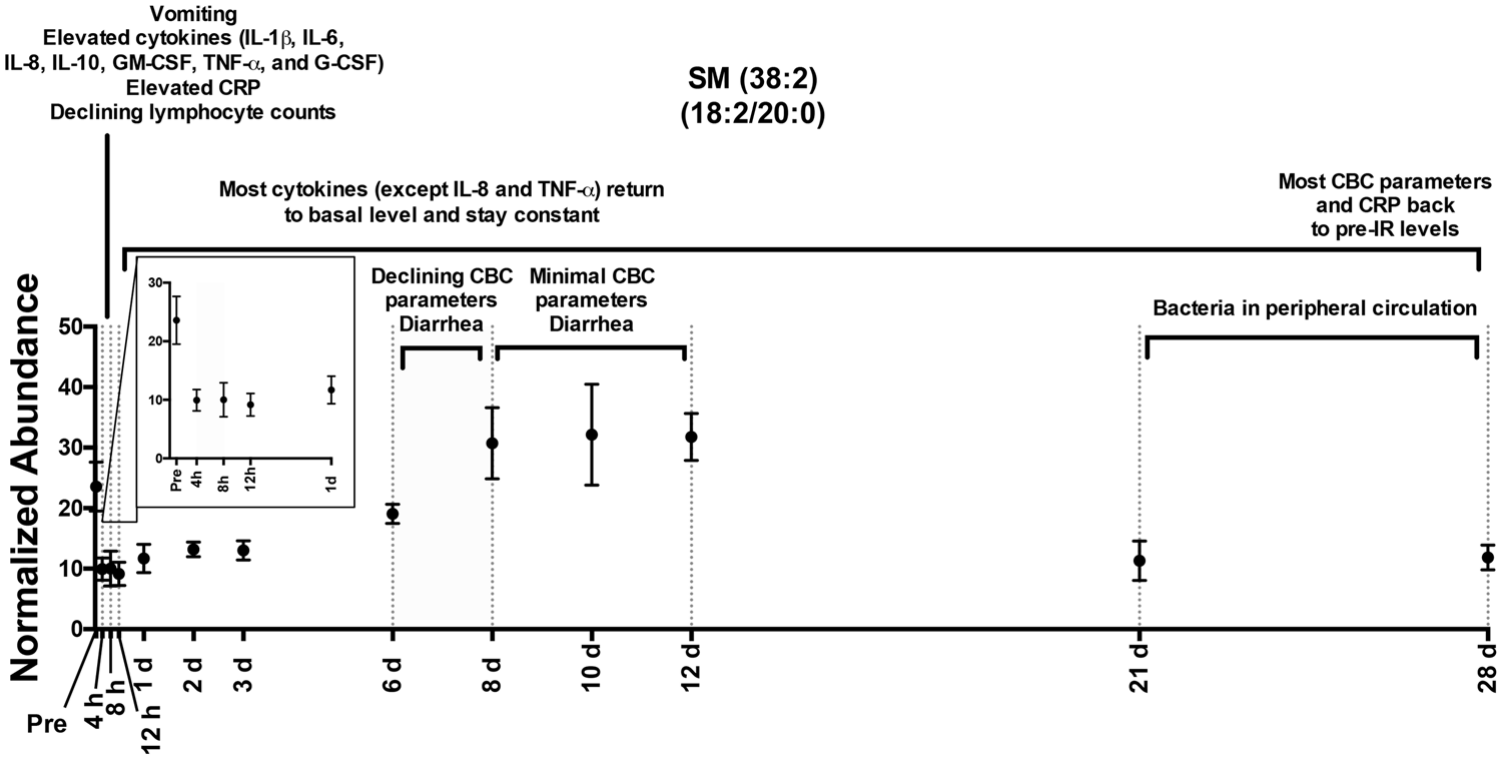
Temporal response of SM (38:2) in the serum of NHPs exposed to 6.5 Gy γ-radiation with x-axis corrected for timeline. SMs decrease from pre-irradiation levels for ~1 week, return to higher concentrations during declining complete blood count (CBC) parameters, and then decrease when bacteria is observed in the peripheral circulation. Inset plot accentuates time points from pre-irradiation to 1 d. Changes in cytokine levels (Supplementary Fig. 8)^15^, CBC parameters (Supplementary Fig. 3)^15^, and CRP levels (Supplementary Fig. 12)^15^ can be found in Singh *et al*.^15^.

Clinical signs of hematopoietic syndrome include possible non-reversible damage to hematopoietic tissues throughout bone marrow, pancytopenia, and cytopenia. Hematologic profiles demonstrated significant hematological injury in all animals, and histopathology of bone marrow with H & E staining of two euthanized animals showed significant bone marrow aplasia, providing evidence that hematopoietic syndrome was induced^15^. The normalized dataset for electrospray ionization (ESI^+^ and ESI^−^) modes were processed through MetaboLyzer and a standard singular value decomposition based principal component analysis (PCA) was performed. Examples of volcano plots, PCA plots, and heatmaps indicate differences between pre-irradiation samples and samples from three time points (4 h, 6 d, 28 d) are provided in Supplementary Fig. S1. Multidimensional scaling (MDS) plots and heatmaps were generated for the top 100 ions with the machine learning algorithm Random Forest (RF) (Figs 1 and 2). For MDS plots, time points were divided to illustrate the first week (4 h, 8 h, 12 h, 1 d, 2 d, 3 d, 6 d) and the remaining time points (6 d, 8 d, 10 d, 12 d, 21 d, 28 d) (Fig. 1). In ESI^+^ mode RF classified the top 100 ions from the first set of time points with 74.6% accuracy and 82.4% for the second group of time points. During the first week, samples separated from the pre-irradiation samples along dimension 2 at 4 h–8 h, while returning closer to pre-irradiation levels at 12 h and 1 d (Fig. 1A). This corresponds to an increase in cytokines and C-reactive protein during this period, which returned to basal levels by 12 h^15^. During 1 d to 6 d data points begin to separate along dimension 1, corresponding to declining complete blood count (CBC) parameters, on set of diarrhea, and hair loss (Figs 1A and 3, Supplementary Figs 2, 3). From 6 d–28 d, pre-irradiation samples separate along dimension 2 from 6 d–8 d and across dimension 1 from 8 d–21 d (Fig. 1B). At 28 d, most CBC parameters and C-reactive protein returned to pre-irradiation levels; however, bacteremia was observed in the peripheral circulation (Supplementary Table S1). The presence of bacteria and recovering CBC parameters may explain high variation at 28 d, as points are scattered across the MDS plot, with values closer to the pre-irradiation samples. The heatmap shows similar trends with a group of ions increasing in intensity up to 1 d, with other ions increasing from 2 d–10 d (Fig. 2). Changes in cytokine levels (Supplementary Fig. 8)^15^, CBC parameters (Supplementary Fig. 3)^15^, and CRP levels (Supplementary Fig. 12)^15^ can be found in Singh *et al*.^15^.

### MS/MS Identification and Univariate Analysis

#### 4 h–1 d

**Figure 4:**
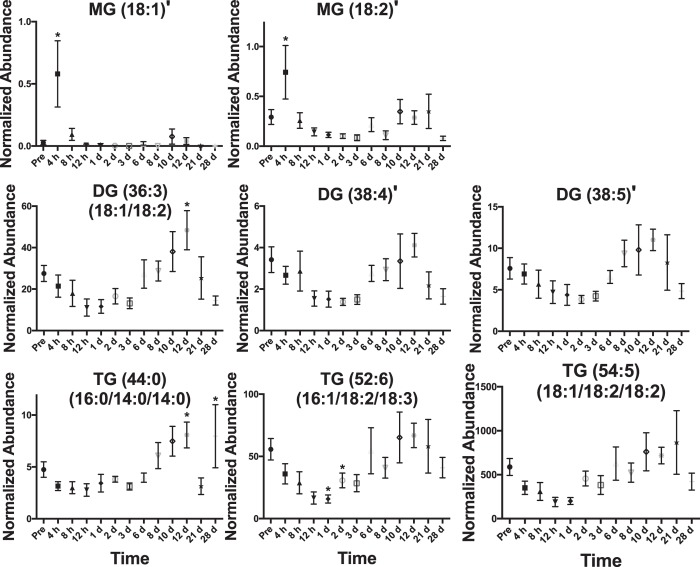
Temporal response of glycerolipids in the serum of NHPs exposed to 6.5 Gy γ-radiation (* with bar signify groups significantly different [*p* < 0.05] from pre-irradiation determined by post-hoc Duncan test). MGs show a significant increase at 4 h. DGs and TGs slightly decrease until ~1 week and increase in concentration. Acyl constituents were determined by tandem MS (‘ indicates concentrations too low for tandem MS).

**Figure 5:**
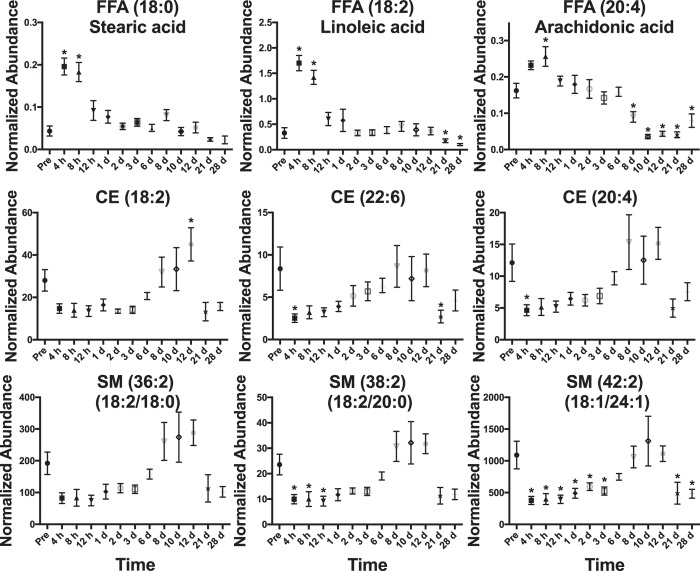
Temporal response of FFAs, CEs, and SMs in the serum of NHPs exposed to 6.5 Gy γ-radiation (*with bar signify groups significantly different [*p* < 0.05] from pre-irradiation determined by post-hoc Duncan test). FFAs increase during the first 8 h return to pre-irradiation concentrations within 24 h, corresponding to increases in cytokine levels. CEs and SMs decrease for ~1 week, then return to higher concentrations. Acyl constituents were determined by tandem MS.

**Figure 6:**
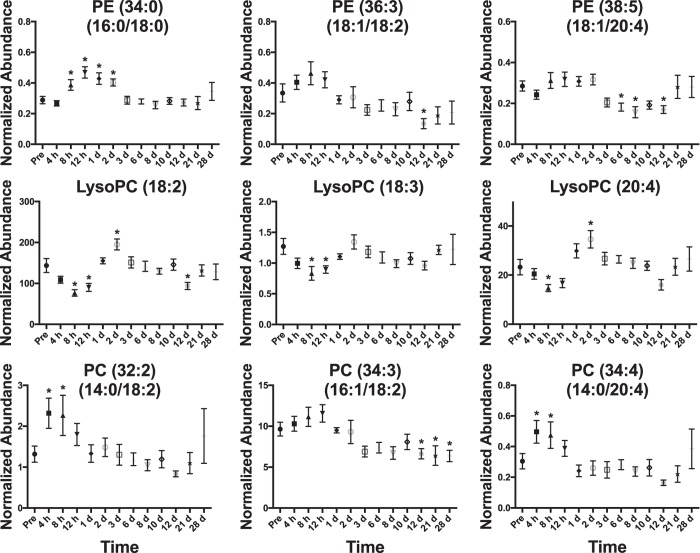
Temporal response of GPs in the serum of NHPs exposed to 6.5 Gy γ-radiation (*with bar signify groups significantly different [*p* < 0.05] from pre-irradiation determined by post-hoc Duncan test). Slight increases are observed in diacyl GPs during the first 24 h while lysoPCs decrease during the first 24 h. Acyl constituents were determined by tandem MS.

A total of 195 significant lipids (*p* < 0.05) were identified through a Kruskal-Wallis test (*p* values and fold changes listed in Supplementary Table S2). During 4 h–8 h, increasing C-reactive protein and select cytokine levels were elevated. Increases in MG concentration at 4 h were observed with levels returning to normal after 8 h (Fig. 4). The observed increases in MG (18:1) and (18:2) may be products from DGs and TGs through increased lipolytic activity or oxidation, which show a slight decrease through this time to 1 d (Fig. 4). Similar to MGs, Stearic acid (FFA 18:0) and linoleic acid (FFA 18:2) significantly increased at 4 h and 8 h and returned to normal levels after 12 h (Fig. 5). Arachidonic acid (FFA 20:4) was significantly higher at 8 h and slightly decreased to 1 d. CEs and SMs decreased throughout this phase (Figs 3 and 5). PC (14:0/18:2) and (14:0/20:4) both significantly increased at 4 h and 8 h and a slight increase in PC (16:1/18:2) was observed, with concentrations returning to normal after 1 d (Fig. 6). LysoPCs decreased during the 1 d time point.

#### 2 d–6 d

A few lipid levels varied from the pre-irradiation time point throughout this time. Small increases for DGs, TG (16:0/14:0/14:0), TG (16:1/18:2/18:3), TG (18:1/18:2/18:2), CE (18:2), CE (22:6), and CE (20:4) were observed during this time point (Figs 4 and 5). SM (18:2/18:0), SM (18:2/20:0), and SM (18:1/24:1) remained lower than the pre-irradiation time point (Figs 3 and 5). LysoPC (18:2) and (20:4) were significantly higher at 2 d and returned to pre-irradiation through this time (Fig. 6).

#### 8 d–28 d

During 8 d–12 d, CBC parameters reached nadir. Increases in DG (18:1/18:2), DG (38:4), DG (38:5), CE (18:2), CE (22:6), CE (20:4), SM (18:2/18:0), SM (18:2/20:0), and SM (18:1/24:1) occur through ~8 d–12 d and then decrease at the 21 d and 28 d time points (Figs 3, 5 and 6). At 21 d and 28 d, several NHPs had bacteria in peripheral circulation. FFA (20:4) significantly decreased through this time period (Fig. 5).

## Discussion

This study reports changes in NHP serum lipidomic signatures spanning 28 d following exposure to 6.5 Gy radiation and how lipid changes correlate with CBC parameters, cytokine levels, and NHP clinical signs (see Fig. 3). Studies have reported altered lipid signatures after γ-radiation exposure or internal emitter exposure in mice^16, 17^, in NHPs at 7 d post-irradiation^13, 14^, and lipid peroxidation from irradiation exposures has been well documented^18, 19^. To our knowledge, this is the first study to specifically determine temporal variation in lipid signatures in NHP biofluids with radiation-induced hematopoietic syndrome. At a 6.5 Gy exposure in NHP, the hematopoietic syndrome will be induced without high interference from gastrointestinal syndrome. Hematopoietic syndrome is marked by radiation-induced possible non-reversible damage to the hematopoietic tissues throughout the bone marrow, stem cell elimination, cytopenia, and possibly pancytopenia. Determining radiation-induced changes to serum lipidomic signatures over time may identify compounds useful for biodosimetry in the event of a nuclear emergency and underlying mechanisms of radiation toxicity. Identifying individuals with hematopoietic syndrome is of particular concern since earlier triage and medical intervention (e.g. cytokine therapy to stem cell transplantation) would greatly increase survival rates. We found perturbations to several lipid species including GPs, GLs, CEs, FFAs, and SMs. The most striking differences were observed within the first 24 h during initial increase in cytokine levels and C-reactive protein, and then after ~1 week during declining CBC parameters^15^. SMs decrease 2–3 d post-irradiation while LysoPCs slightly increase at 2 d, making these compounds possible markers for biodosimetry. At higher doses (10 Gy), previous reports indicate LysoPCs remain elevated up to 7 d post-irradiation^13^. Early upregulated ions (<1 d) may represent inflammatory lipids increasing in concentration with increased cytokine levels, while other lipids increasing from 2 d–10 d represent lipids corresponding to declining CBC parameters. At the 21 d and 28 d time points bacteria was observed in the peripheral circulation, indicating possible GI leakage and may contribute to higher variability observed in the NHP serum lipidome (Supplementary Table S1) as well as the host response to LPS and other bacterial products.

After radiation exposure, an individual will initially experience nausea/emesis, diarrhea, fever, or headaches^20, 21^. A 6.5 Gy exposure in NHPs is approximately similar to a 3.5 Gy equitoxic dose for humans. It is well-established that in addition to direct damage to DNA following radiation exposure (e.g., single- and double-stranded breaks, DNA crosslinking), radiation reacts with water molecules producing ROS, which in turn react with cellular proteins and lipid molecules among others, damaging these substrates and producing an inflammatory response directly or indirectly. ROS reactions with lipids affect the molecular integrity by both peroxidation of hydrophobic portions or free-radical cleavage of parent molecules, and molecules may go through hydrolytic cleavage of ester, ether, and amide bonds (e.g., lipolysis through enzymes and hormones)^22–24^. The serum lipidome reflects acute changes in specific lipid molecular concentration during early post-irradiation. Specifically, early increases in MG, PC, and FFAs occur (at 4 h for MG, through 8 h for FFAs, and at 4 h and 8 h for PC [32:2] and [34:4]) with decreases in concentration of DGs, TGs, CEs, LysoPCs, and SMs. Increases in concentration of MG may be due to oxidation and hydrolytic processes of parent glycerolipids DG and TG molecules, which decrease in concentration during the first 24 h, or possibly be diet related due to radiation induced emesis or weight loss (Supplementary Table S3). Hydrolytic cleavage of acyl constituents may also occur for glycerolipids and CEs, as these storage/transport molecules may transport FFAs such as (18:2) and (20:4) to sites of tissue damage to initiate inflammation via the COX and LOX pathways. Both the lipolytic and peroxide products produced through ROS generation after irradiation exposure contribute to inflammation observed and may provide positive feedback mechanisms further producing ROS^5, 6^.

SMs have been extensively studied after radiation exposure due to their ability to undergo free-radical fragmentation of the amide bond and more importantly to form ceramides involved in apoptosis^25, 26^. In this study, SMs decrease ~4 h–3 d, making them possible candidates for biodosimetry. SMs are synthesized primarily in the Golgi apparatus through the catalysis of PCs to ceramides by sphingomyelin synthase (EC: 2.7.8.27), thus producing SMs and DGs. Sphingomyelinases (EC: 3.1.4.12) enzymatically convert SMs to ceramides and phosphorycholine or fragment to yield a fatty acid amide^22, 27^. Further metabolism of SM yields many compounds, some with bioactive properties, such as sphingosines, galactoceramide, and ceramides phosphates. Although SM concentration is visibly affected after radiation exposure, ceramides were not detected in the current analysis and may require targeted approaches or other methods^28^ to determine if ceramide production is upregulated. Interestingly, SM containing a 24:1 (nervonic acid) acyl was reduced, which is an important central nervous system (CNS) structural lipid. Decreases in 24:1 may be due to a decrease in biosynthesis from 18:1 (oleic acid) as seen in demyelinating diseases^29^ or oxidation from free radicals.

Lipidomics is a subfield within metabolomics that is ideal for generating large and informative datasets to determine changes in tissues and fluids after toxin exposure, disease states, and try to identify useful and accurate biomarkers^4^. Considerable interest has been dedicated to determining if metabolomics and lipidomics can aid in biodosimetry after nuclear emergencies^1, 2^. While previous studies have implicated changes in the lipidome after radiation exposure^13, 14, 16, 28, 30^, few have included a temporal description of lipid perturbation^17, 31^, which were restricted to internal emitter exposure in a murine model. Here, we provide an example of the complexity of lipid responses in a NHP model to irradiation over 4 h–28 d. Future studies may expand radiation lipidomics beyond 28 d to determine markers of DEARE.

## Methods

### Chemicals

Standards for lipidomics included SM (d18:1/12:0), PE (14:0/14:0), PC (14:0/14:0), PS (14:0/14:0), PI (17:0/20:4), LPC (17:1), CE (19:0) (Avanti Polar Lipids, Inc., Alabaster, AL) and TG (19:1/19:1/19:1), DG (20:1/20:1), MG (17:1), FFA (17:1) (Nu-check Prep Inc., Elysian, MN). Reagents were Fisher Scientific Optima^TM^ LC/MS grade (Hanover Park, IL) and all standards were of the highest purity available.

### Nonhuman Primate Model, Experimental Treatments, and Biofluid Collection

Serum samples were obtained from an earlier study for which details on materials and methods, and serum collection have been previously described^15^. Briefly, rhesus NHPs (*Macaca mulatta*, Chinese sub strain, Primate Products, Inc., Miami, FL, USA) were quarantined (6–7 weeks prior to the experiment) and maintained in a facility accredited by the Association for Assessment and Accreditation of Laboratory Animal Care (AAALAC)-International. Healthy male and female individuals (8 animals for current study [4 male, 4 female], 3–4 yrs old, 3.8–5.3 kg) were used in this study. NHPs were housed in stainless-steel cages (1 animal per cage, 22 °C ± 2 °C, 30–70% relative humidity, 12 h light:12 h dark cycle, and 10–15 air change cycles per h), fed primate chow (Harlan Teklad Global 20% Protein Primate T.2050 diet, Madison, WI, USA) twice daily, received enrichment food (fresh fruits/vegetables, etc.) once a day during weekdays, and received drinking water *ad libitum*. All procedures involving animals were approved by the Institutional Animal Care and Use Committee (IACUC) and Department of Defense Animal Care and Use Review Office (ACURO), and in strict accordance with published recommendations^32^.

Food was withheld from each animal (~12–18 h) prior to radiation exposure (to minimize radiation-induced vomiting). NHPs were administered ketamine hydrochloride (10–15 mg/kg, ~30–45 min prior) intramuscularly, secured in irradiation chambers, and exposed with a midline dose of 0.6 Gy/min (6.5 Gy total). Dose rate measurements were based primarily on the alanine/EPR (electron paramagnetic resonance) system as previously described^15^. Calibration curves (EMXmicro spectrometer, Bruker Corp., Billerica, MA, USA) used were based on standard alanine calibration sets (National Institute of Standards and Technology, Gaithersburg, MD, USA).

Blood was collected by venipuncture from the saphenous vein on the caudal aspect of the lower leg, placed in serum separating tubes, allowed to clot for 30 min, and centrifuged (10 min, 400 × *g*). Two NHPs (one female and one male) were euthanized on day 17 due to moribundity. Serum samples were stored at −70 °C until shipped on dry ice to the Georgetown University Medical Center.

### Sample Preparation and Analysis

Lipid extraction was performed as previously described^13^. Briefly, serum samples (25 μl) were extracted with solvent (100 μl, 2:1 cold chloroform:methanol) containing internal standards (0.5 μM each), vortexed, incubated for 5 min (room temperature), vortexed, and centrifuged for phase separation (10,000 × *g*, 4 °C, 10 min). The lower organic phase was collected, evaporated under N_2_, and reconstituted (200 μl, 50:25:25 isopropanol:acetonitrile:H_2_O). Samples were injected (1 μl) into an Acquity UPLC H-Class (Waters Corporation, MA) equipped with a CSH C18 1.7 μm, 2.1 × 100 mm column. The UPLC system was coupled to a Synapt^®^ G2-Si HDMS QTOF-MS and analyzed in the positive and negative mode using chromatographic conditions as previously described^33^. Leucine enkephalin (556.2771 [M + H]^+^ or 554.2615 [M−H]^−^) was used for accurate mass calibration as Lockspray^®^.

### Data Processing, Statistical Analysis, and Marker Validation

Total ion chromatograms (TIC) were processed in Progenesis QI (Nonlinear Dynamics, Newcastle, UK) using settings previously described^13^. Samples were aligned to pooled quality control samples with the highest similarity being chosen by the software. Aligned centroided samples were putatively identified by searching the LIPID MAPS database and MetaScope theoretical fragment search^8, 9^. Ions of interest were normalized to their respective internal standard, analyzed with a Kruskal-Wallis test, and finally analyzed with a post-hoc Duncan test (SAS 9.4, Cary, NC). The machine-learning algorithm RF was applied through the programming language R producing a MDS and heatmap of the top 100 metabolites, as ranked through RF. A heatmap and volcano plot were generated with the software MetaboLyzer on complete-presence ions (with 10 ppm error cut-off), which are defined as ions that are present in at least 70% of the samples in both analysis groups^34^. A standard singular value decomposition based PCA was performed. Select compounds were identified to molecular species by tandem MS using a ramping collision energy from 5–50 V.

## Electronic supplementary material

Supplementary Information
