# Author Correction: Lipidomic Signatures of Nonhuman Primates with Radiation-Induced Hematopoietic Syndrome

**DOI:** 10.1038/s41598-019-42491-5

**Published:** 2019-05-03

**Authors:** Evan L. Pannkuk, Evagelia C. Laiakis, Vijay K. Singh, Albert J. Fornace

**Affiliations:** 1 Tumor Biology Program, Lombardi Comprehensive Cancer Center, Georgetown University, Washington, D.C. 20057 USA; 2 Department of Biochemistry and Molecular & Cellular Biology, Georgetown University Medical Center, Washington, D.C. 20057 USA; 3 Department of Pharmacology and Molecular Therapeutics, F. Edward Hébert School of Medicine, Bethesda, USA; 4 Armed Forces Radiobiology Research Institute, Uniformed Services University of the Health Sciences, Bethesda, MD 20814 USA; 5 Deparment of Oncology Georgetown University, Washington, D.C. 20057 USA

Correction to: *Scientific Reports* 10.1038/s41598-017-10299-w, published online 29 August 2017

The original version of this Article contained an error in the Abstract.

“The current study addresses temporal changes in the serum lipidome from 4 h to 28 d in nonhuman primates (NHPs) with radiation-induced hematopoietic syndrome (6.5 Gy exposure, LD_50/30_).”

now reads:

“The current study addresses temporal changes in the serum lipidome from 4 h to 28 d in nonhuman primates (NHPs) with radiation-induced hematopoietic syndrome (6.5 Gy exposure, LD_50/60_).”

This error has now been corrected in the PDF and HTML versions of the Article.

