# Supplementary material for: Lipidomic Signatures of Nonhuman Primates with Radiation-Induced Hematopoietic Syndrome
Source: Sci Rep. 2017 Aug 29;7:9777. doi: 10.1038/s41598-017-10299-w (PMC5575047; doi:10.1038/s41598-017-10299-w)
Supplement: Supplementary file 1 — Supplementary Information [file 41598_2017_10299_MOESM1_ESM.pdf]

Lipidomic Signatures of Nonhuman Primates with Radiation-Induced Hematopoietic Syndrome

Evan L. Pannkuk,[1] Evagelia C. Laiakis,[2] Vijay K. Singh,[3,4] and Albert J. Fornace Jr.[2,5,*]

[1]Tumor Biology Program, Lombardi Comprehensive Cancer Center, Georgetown University, Washington, D.C., 20057, U.S.A. [2]Department of Biochemistry and Molecular & Cellular Biology, Georgetown University Medical Center, Washington, D.C., 20057, U.S.A. [3]Department of Pharmacology and **Molecular** Therapeutics, F. Edward Hébert School of Medicine, [4]Armed Forces Radiobiology Research Institute, Uniformed Services University of the Health Sciences, Bethesda, MD, 20814, U.S.A. **[5]Deparment of Oncology Georgetown University, Washington, D.C., 20057, U.S.A.** [*]Correspondence and request for materials should be addressed to A.F. (af294@georgetown.edu)

Supplementary Table S1. Bacteria isolated in peripheral blood samples of animals (5 out of 8 had bacteria).

NHP #	Specimen	SD	Gram stain Result	Identification
805356F	blood	17	Gram positive, cocci, clusters	*Staphylococcus auerus*
	blood	24	Gram positive, cocci, clusters	*Staphylococcus warneri*
807123M	blood	8	Gram positive, cocci, clusters	*Staphylococcus warneri*
	blood	17	Gram positive, cocci, clusters	*Staphylococcus warneri*
	blood	42	Gram positive, cocci, chains and clusters	*Staphylococcus auricularis*
		51	Gram positive, cocci, clusters	*Staphylococcus warneri*
805093M	blood	17	Gram negative, rods	*Escherichia coli*
805115M	blood	17	Gram positive, cocci, pair and clusters dark stain	*Staphylococcus warneri*
	blood	17	Gram positive, cocci, honey comb like pattern	*Streptococcus uberic*
	blood	24	Gram positive, cocci, clusters	*Kocuria kristinae*
	blood	24	Gram positive, cocci, clusters	*Staphylococcus warneri*
806428F	blood	17	gram positive, cocci, clusters, dark stain	*Stephylococcus warneri*
	blood	17	Gram negative, chains elongated but not quite like rods	*Sphmon. paucimobilis*

Supplementary Table S2. Comprehensive list of lipids that significantly changed after exposure to ionizing radiation exposure (Adducts NH_4^+=TG, DG, CE; Na^+=TG, DG, MG, CE; H^+=TG, MG, SM, PC, ePC, LysoPC; H^-=FFA, PE, ePE, LysoPE, PS; Kruskal-Wallis test, $P < 0.05$). (*m/z*=mass-to-charge ratio, rt=retention time, MFC=max fold change, HM=highest mean, LM=lowest mean)

Broad Class	Sub-Class	*m/z*_rt	Compound	Formula	MFC	HM	LM	p-value
**Glycerolipids**	**Triacylglycerides**	689.57277_6.77	TG(38:0)	C41H78O6	1.7	28 d	2 d	<0.0001
**Glycerolipids**	**Triacylglycerides**	687.55400_6.53	TG(38:1)	C41H76O6	1.8	28 d	3 d	<0.0001
**Glycerolipids**	**Triacylglycerides**	712.64435_7.03	TG(40:0)	C43H82O6	2.3	28 d	2 d	<0.0001
**Glycerolipids**	**Triacylglycerides**	715.57843_6.81	TG(40:1)	C43H80O6	7.9	28 d	8 hr	0.0065
**Glycerolipids**	**Triacylglycerides**	740.67577_7.26	TG(42:0)	C45H86O6	3.1	28 d	8 hr	<0.0001
**Glycerolipids**	**Triacylglycerides**	738.65943_7.06	TG(42:1)	C45H84O6	6.5	28 d	8 hr	<0.0001
**Glycerolipids**	**Triacylglycerides**	768.70617_7.45	TG(44:0)	C47H90O6	4.5	28 d	12 hr	<0.0001
**Glycerolipids**	**Triacylglycerides**	766.69048_7.26	TG(44:1)	C47H88O6	5.3	28 d	8 hr	0.0003
**Glycerolipids**	**Triacylglycerides**	764.67496_7.08	TG(44:2)	C47H86O6	12.4	28 d	2 d	0.0083
**Glycerolipids**	**Triacylglycerides**	796.73830_7.63	TG(46:0)	C49H94O6	3.2	28 d	8 d	<0.0001
**Glycerolipids**	**Triacylglycerides**	794.72320_7.45	TG(46:1)	C49H92O6	6.1	28 d	8 d	0.0047
**Glycerolipids**	**Triacylglycerides**	824.77023_7.80	TG(48:0)	C51H98O6	1.8	28 d	8 d	0.0102
**Glycerolipids**	**Triacylglycerides**	822.75545_7.63	TG(48:1)	C51H96O6	3.6	28 d	8 d	0.0072
**Glycerolipids**	**Triacylglycerides**	820.73980_7.47	TG(48:2)	C51H94O6	3.7	28 d	12 hr	0.0030
**Glycerolipids**	**Triacylglycerides**	818.72358_7.32	TG(48:3)	C51H92O6	4.1	28 d	8 d	0.0324
**Glycerolipids**	**Triacylglycerides**	846.75512_7.49	TG(50:3)	C53H96O6	2.3	6 d	1 d	0.0029
**Glycerolipids**	**Triacylglycerides**	844.73994_7.34	TG(50:4)	C53H94O6	2.5	21 d	1 d	0.0007
**Glycerolipids**	**Triacylglycerides**	883.76856_7.80	TG(52:1)	C55H104O6	2.8	21 d	1 d	0.0008
**Glycerolipids**	**Triacylglycerides**	874.78729_7.67	TG(52:3)	C55H100O6	3.1	21 d	12 hr	0.0009
**Glycerolipids**	**Triacylglycerides**	872.77188_7.52	TG(52:4)	C55H98O6	3.1	21 d	12 hr	0.0006
**Glycerolipids**	**Triacylglycerides**	870.75576_7.38	TG(52:5)	C55H96O6	3.1	21 d	12 hr	0.0002
**Glycerolipids**	**Triacylglycerides**	868.73865_7.21	TG(52:6)	C55H94O6	2.8	21 d	1 d	0.0009
**Glycerolipids**	**Triacylglycerides**	911.79639_7.96	TG(54:1)	C57H108O6	2.8	21 d	8 d	0.0022

**Glycerolipids**	**Triacylglycerides**	907.77423_7.83	TG(54:3)	C57H104O6	2.5	21 d	1 d	0.0108
**Glycerolipids**	**Triacylglycerides**	900.80232_7.69	TG(54:4)	C57H102O6	3.0	21 d	1 d	0.0025
**Glycerolipids**	**Triacylglycerides**	898.78676_7.54	TG(54:5)	C57H100O6	3.1	21 d	1 d	0.0012
**Glycerolipids**	**Triacylglycerides**	896.77148_7.38	TG(54:6)	C57H98O6	3.4	21 d	12 hr	0.0003
**Glycerolipids**	**Triacylglycerides**	894.75553_7.24	TG(54:7)	C57H96O6	3.4	21 d	1 d	0.0002
**Glycerolipids**	**Triacylglycerides**	892.73950_7.08	TG(54:8)	C57H94O6	3.1	21 d	1 d	0.0004
**Glycerolipids**	**Triacylglycerides**	891.65552_6.13	TG(54:11)	C57H88O6	1.8	Pre-IR	21 d	<0.0001
**Glycerolipids**	**Triacylglycerides**	934.87946_8.24	TG(56:1)	C59H112O6	6.8	21 d	12 hr	0.0001
**Glycerolipids**	**Triacylglycerides**	932.86443_8.11	TG(56:2)	C59H110O6	6.7	21 d	12 hr	0.0002
**Glycerolipids**	**Triacylglycerides**	930.84869_7.98	TG(56:3)	C59H108O6	4.1	21 d	1 d	0.0007
**Glycerolipids**	**Triacylglycerides**	928.83235_7.85	TG(56:4)	C59H106O6	3.3	21 d	8 d	0.0030
**Glycerolipids**	**Triacylglycerides**	926.81493_7.78	TG(56:5)	C59H104O6	3.8	21 d	8 d	0.0027
**Glycerolipids**	**Triacylglycerides**	922.78611_7.47	TG(56:7)	C59H100O6	3.4	21 d	8 d	0.0128
**Glycerolipids**	**Triacylglycerides**	920.77114_7.34	TG(56:8)	C59H98O6	3.5	21 d	8 d	0.0125
**Glycerolipids**	**Triacylglycerides**	918.75582_7.19	TG(56:9)	C59H96O6	3.1	21 d	8 d	0.0030
**Glycerolipids**	**Triacylglycerides**	916.74211_7.03	TG(56:10)	C59H94O6	2.9	21 d	8 d	0.0046
**Glycerolipids**	**Triacylglycerides**	962.91014_8.35	TG(58:1)	C61H116O6	4.0	21 d	8 d	<0.0001
**Glycerolipids**	**Triacylglycerides**	960.88789_8.24	TG(58:2)	C61H114O6	8.9	21 d	4 hr	0.0001
**Glycerolipids**	**Triacylglycerides**	958.88021_8.11	TG(58:3)	C61H112O6	11.8	21 d	4 hr	0.0001
**Glycerolipids**	**Triacylglycerides**	956.86326_8.00	TG(58:4)	C61H110O6	13.8	21 d	1 d	0.0004
**Glycerolipids**	**Triacylglycerides**	952.83330_7.76	TG(58:6)	C61H106O6	4.3	21 d	8 d	0.0012
**Glycerolipids**	**Triacylglycerides**	950.81792_7.61	TG(58:7)	C61H104O6	3.7	21 d	8 d	0.0011
**Glycerolipids**	**Triacylglycerides**	948.80236_7.56	TG(58:8)	C61H102O6	4.0	21 d	8 d	0.0022
**Glycerolipids**	**Triacylglycerides**	940.73084_6.97	TG(58:12)	C61H94O6	7.3	21 d	12 d	0.0033
**Glycerolipids**	**Triacylglycerides**	986.90907_8.24	TG(60:3)	C63H116O6	6.8	21 d	1 d	0.0007
**Glycerolipids**	**Triacylglycerides**	984.89584_8.13	TG(60:4)	C63H114O6	15.7	21 d	12 hr	0.0003
**Glycerolipids**	**Triacylglycerides**	982.87724_8.00	TG(60:5)	C63H112O6	36.7	21 d	8 d	0.0079
**Glycerolipids**	**Triacylglycerides**	980.86031_7.91	TG(60:6)	C63H110O6	8.8	21 d	8 d	0.0295

**Glycerolipids**	**Triacylglycerides**	978.84557_7.76	TG(60:7)	C63H108O6	4.3	21 d	8 hr	0.0004
**Glycerolipids**	**Triacylglycerides**	974.81733_7.59	TG(60:9)	C63H104O6	4.9	21 d	8 d	0.0010
**Glycerolipids**	**Triacylglycerides**	998.81619_7.47	TG(62:11)	C65H104O6	47.2	21 d	8 d	0.0004
**Glycerolipids**	**Triacylglycerides**	968.77066_7.21	TG(60:12)	C63H98O6	8.1	21 d	8 d	0.0187
**Glycerolipids**	**Diacylglycerides**	591.49552_6.07	DG(32:0)	C35H68O5	2.9	4 hr	8 d	0.0394
**Glycerolipids**	**Diacylglycerides**	619.52689_6.40	DG(34:0)	C37H72O5	2.0	12 d	8 d	0.0006
**Glycerolipids**	**Diacylglycerides**	617.51338_6.11	DG(34:1)	C37H70O5	2.3	12 d	1 d	0.0008
**Glycerolipids**	**Diacylglycerides**	615.49589_5.83	DG(34:2)	C37H68O5	2.2	4 hr	2 d	0.0005
**Glycerolipids**	**Diacylglycerides**	647.55832_6.68	DG(36:0)	C39H76O5	1.5	12 d	Pre-IR	<0.0001
**Glycerolipids**	**Diacylglycerides**	645.54898_6.40	DG(36:1)	C39H74O5	3.0	12 d	8 d	0.0062
**Glycerolipids**	**Diacylglycerides**	643.52902_6.15	DG(36:2)	C39H72O5	2.0	12 d	1 d	0.0003
**Glycerolipids**	**Diacylglycerides**	641.51227_5.87	DG(36:3)	C39H70O5	2.0	4 hr	1 d	0.0002
**Glycerolipids**	**Diacylglycerides**	639.49783_5.58	DG(36:4)	C39H68O5	3.6	4 hr	12 hr	<0.0001
**Glycerolipids**	**Diacylglycerides**	669.54741_6.26	DG(38:3)	C41H74O5	4.6	Pre-IR	2 d	0.0044
**Glycerolipids**	**Diacylglycerides**	667.52785_6.11	DG(38:4)	C41H72O5	2.7	4 hr	2 d	0.0031
**Glycerolipids**	**Diacylglycerides**	665.50821_5.80	DG(38:5)	C41H70O5	2.6	4 hr	2 d	0.0040
**Glycerolipids**	**Diacylglycerides**	699.59055_6.71	DG(40:2)	C43H80O5	1.4	6 d	2 d	<0.0001
**Glycerolipids**	**Diacylglycerides**	732.54923_6.81	DG(44:11)	C47H70O5	1.5	12 d	2 d	<0.0001
**Glycerolipids**	**Monoacylglycerides**	379.28135_2.40	MG(18:1)	C21H40O4	-	4 hr	3 d	0.0005
**Glycerolipids**	**Monoacylglycerides**	377.26573_1.85	MG(18:2)	C21H38O4	12.3	4 hr	8 d	0.0034
**Sterols**	**Cholesteryl Esters**	671.57361_7.73	CE(18:2)	C45H76O2	1.8	12 d	2 d	<0.0001
**Sterols**	**Cholesteryl Esters**	664.60054_7.59	CE(18:3)	C45H74O2	1.7	4 hr	2 d	0.0003
**Sterols**	**Cholesteryl Esters**	695.57680_7.65	CE(20:4)	C47H76O2	1.7	6 d	21 d	0.0002
**Sterols**	**Cholesteryl Esters**	719.57216_7.56	CE(22:6)	C49H76O2	2.0	6 d	21 d	0.0062
**Free Fatty Acids**		255.23254_2.79	FFA 16:0	C16H32O2	14.0	4 hr	28 d	<0.0001
**Free Fatty Acids**		253.21678_2.16	FFA 16:1	C16H30O2	-	4 hr	21 d	<0.0001
**Free Fatty Acids**		283.26375_3.59	FFA 18:0	C18H36O2	9.9	4 hr	28 d	<0.0001
**Free Fatty Acids**		281.24825_2.90	FFA 18:1	C18H34O2	32.6	4 hr	28 d	<0.0001

**Free Fatty Acids**		279.23250_2.36	FFA 18:2	C18H32O2	18.9	4 hr	28 d	<0.0001
**Free Fatty Acids**		303.23237_2.24	FFA 20:4	C20H32O2	7.2	8 hr	10 d	<0.0001
**Sphingolipids**	**Sphingomyelin**	677.55847_4.39	SM(32:0)	C37H77N2O6P	1.7	12 hr	8 d	<0.0001
**Sphingolipids**	**Sphingomyelin**	675.54379_4.18	SM(32:1)	C37H75N2O6P	1.5	12 hr	2 d	<0.0001
**Sphingolipids**	**Sphingomyelin**	673.52719_3.69	SM(32:2)	C37H73N2O6P	1.7	8 hr	12 d	0.0011
**Sphingolipids**	**Sphingomyelin**	703.57568_4.72	SM(34:1)	C39H79N2O6P	1.3	28 d	4 hr	<0.0001
**Sphingolipids**	**Sphingomyelin**	701.55935_4.29	SM(34:2)	C39H77N2O6P	1.5	6 d	4 hr	0.0001
**Sphingolipids**	**Sphingomyelin**	731.60726_5.19	SM(36:1)	C41H83N2O6P	1.4	6 d	21 d	<0.0001
**Sphingolipids**	**Sphingomyelin**	729.59134_4.82	SM(36:2)	C41H81N2O6P	1.5	12 d	8 hr	<0.0001
**Sphingolipids**	**Sphingomyelin**	727.56810_4.44	SM(36:3)	C41H79N2O6P	3.2	1 d	28 d	0.0004
**Sphingolipids**	**Sphingomyelin**	759.63288_6.03	SM(38:1)	C43H87N2O6P	5.6	10 d	28 d	<0.0001
**Sphingolipids**	**Sphingomyelin**	757.61953_5.27	SM(38:2)	C43H85N2O6P	1.5	6 d	21 d	<0.0001
**Sphingolipids**	**Sphingomyelin**	811.66425_6.33	SM(40:0)	C45H93N2O6P	1.8	10 d	28 d	0.0001
**Sphingolipids**	**Sphingomyelin**	809.65099_5.98	SM(40:1)	C45H91N2O6P	1.4	6 d	4 hr	<0.0001
**Sphingolipids**	**Sphingomyelin**	807.63374_5.70	SM(40:2)	C45H89N2O6P	1.4	6 d	2 d	<0.0001
**Sphingolipids**	**Sphingomyelin**	815.70151_6.31	SM(42:1)	C47H95N2O6P	1.5	Pre-IR	4 hr	<0.0001
**Sphingolipids**	**Sphingomyelin**	813.68584_5.98	SM(42:2)	C47H93N2O6P	1.4	6 d	4 hr	<0.0001
**Sphingolipids**	**Sphingomyelin**	811.66202_5.70	SM(42:3)	C47H91N2O6P	1.3	12 hr	21 d	<0.0001
**Sphingolipids**	**Sphingomyelin**	843.73036_6.59	SM(44:1)	C49H99N2O6P	1.7	6 d	21 d	<0.0001
**Sphingolipids**	**Sphingomyelin**	841.71100_6.29	SM(44:2)	C49H97N2O6P	1.9	Pre-IR	21 d	<0.0001
**Glycerophospholipid**	**Phosphatidylcholine (PC)**	650.47551_3.65	PC(26:0)	C34H68NO8P	-	4 hr	Pre-IR	0.2699
**Glycerophospholipid**	**Phosphatidylcholine (PC)**	678.50757_4.25	PC(28:0)	C36H72NO8P	1.6	12 hr	2 d	0.0038
**Glycerophospholipid**	**Phosphatidylcholine (PC)**	706.54416_4.77	PC(30:0)	C38H76NO8P	3.0	28 d	12 d	0.0321
**Glycerophospholipid**	**Phosphatidylcholine (PC)**	704.52478_4.35	PC(30:1)	C38H74NO8P	64.6	28 d	12 d	0.0305
**Glycerophospholipid**	**Phosphatidylcholine (PC)**	730.53910_4.49	PC(32:2)	C40H76NO8P	3.5	4 hr	12 d	0.0022
**Glycerophospholipid**	**Phosphatidylcholine (PC)**	728.52218_4.13	PC(32:3)	C40H74NO8P	-	28 d	1 d	0.3670
**Glycerophospholipid**	**Phosphatidylcholine (PC)**	762.60091_5.62	PC(34:0)	C42H84NO8P	1.5	12 hr	4 hr	0.0033
**Glycerophospholipid**	**Phosphatidylcholine (PC)**	760.58634_5.30	PC(34:1)	C42H82NO8P	1.5	12 hr	Pre-IR	0.0073

**Glycerophospholipid**	**Phosphatidylcholine (PC)**	758.57093_4.99	PC(34:2)	C42H80NO8P	1.4	12 hr	Pre-IR	0.0220
**Glycerophospholipid**	**Phosphatidylcholine (PC)**	756.55513_4.64	PC(34:3)	C42H78NO8P	2.1	12 hr	12 d	0.0001
**Glycerophospholipid**	**Phosphatidylcholine (PC)**	754.53812_4.42	PC(34:4)	C42H76NO8P	3.7	4 hr	12 d	0.0008
**Glycerophospholipid**	**Phosphatidylcholine (PC)**	782.57066_4.92	PC(36:4)	C44H80NO8P	1.5	12 hr	12 d	0.0070
**Glycerophospholipid**	**Phosphatidylcholine (PC)**	780.55490_4.62	PC(36:5)	C44H78NO8P	1.7	12 hr	8 d	0.0003
**Glycerophospholipid**	**Phosphatidylcholine (PC)**	778.53953_4.31	PC(36:6)	C44H76NO8P	2.9	4 hr	12 d	0.0227
**Glycerophospholipid**	**Phosphatidylcholine (PC)**	840.65024_6.13	PC(38:0)	C46H92NO8P	8.8	12 hr	12 d	0.0023
**Glycerophospholipid**	**Phosphatidylcholine (PC)**	806.57061_4.82	PC(38:6)	C46H80NO8P	1.5	12 hr	21 d	0.0110
**Glycerophospholipid**	**Phosphatidylcholine (PC)**	868.67030_6.07	PC(40:0)	C48H96NO8P	5.3	21 d	28 d	0.0008
**Glycerophospholipid**	**Phosphatidylcholine (PC)**	842.66406_6.09	PC(40:2)	C48H92NO8P	4.9	10 d	28 d	0.0003
**Glycerophospholipid**	**Phosphatidylcholine (PC)**	862.62597_5.83	PC(40:3)	C48H90NO8P	4.4	Pre-IR	28 d	0.0197
**Glycerophospholipid**	**Phosphatidylcholine (PC)**	832.58578_4.88	PC(40:7)	C48H82NO8P	2.0	12 hr	28 d	0.0251
**Glycerophospholipid**	**Phosphatidylcholine (PC)**	830.57021_4.53	PC(40:8)	C48H80NO8P	2.3	3 d	28 d	0.0003
**Glycerophospholipid**	**Phosphatidylcholine (PC)**	826.53912_4.39	PC(40:10)	C48H76NO8P	3.2	8 hr	28 d	0.0026
**Glycerophospholipid**	**Phosphatidylcholine (PC)**	870.69436_6.40	PC(42:2)	C50H96NO8P	5.5	10 d	28 d	0.0003
**Glycerophospholipid**	**Phosphatidylcholine (PC)**	864.64784_5.67	PC(42:5)	C50H90NO8P	5.3	12 hr	21 d	0.0002
**Glycerophospholipid**	**Phosphatidylcholine (PC)**	862.62832_5.32	PC(42:6)	C50H88NO8P	3.6	12 hr	21 d	0.0224
**Glycerophospholipid**	**Phosphatidylcholine (PC)**	860.61354_5.27	PC(42:7)	C50H86NO8P	2.1	10 d	2 d	0.0049
**Glycerophospholipid**	**Phosphatidylcholine (PC)**	858.59974_4.95	PC(42:8)	C50H84NO8P	2.4	12 hr	28 d	0.0006
**Glycerophospholipid**	**Phosphatidylcholine (PC)**	856.58426_4.62	PC(42:9)	C50H82NO8P	5.4	6 d	28 d	0.0074
**Glycerophospholipid**	**Phosphatidylcholine (PC)**	854.57062_4.44	PC(42:10)	C50H80NO8P	3.3	3 d	28 d	0.0003
**Glycerophospholipid**	**LysoPC**	520.34009_1.13	LysoPC(18:2)	C26H50NO7P	2.3	2 d	8 hr	<0.0001
**Glycerophospholipid**	**LysoPC**	518.32443_0.93	LysoPC(18:3)	C26H48NO7P	1.8	12 d	8 hr	0.0197
**Glycerophospholipid**	**LysoPC**	546.35622_1.26	LysoPC(20:3)	C28H52NO7P	1.6	12 d	8 hr	0.0013
**Glycerophospholipid**	**LysoPC**	544.33952_1.08	LysoPC(20:4)	C28H50NO7P	2.1	2 d	8 hr	<0.0001
**Glycerophospholipid**	**LysoPC**	542.32195_0.70	LysoPC(20:5)	C28H48NO7P	-	Pre-IR	10 d	0.0006
**Glycerophospholipid**	**LysoPC**	572.36935_1.41	LysoPC(22:4)	C30H54NO7P	1.6	1 d	21 d	0.0025
**Glycerophospholipid**	**LysoPC**	570.35460_1.15	LysoPC(22:5)	C30H52NO7P	1.8	1 d	8 hr	<0.0001

**Glycerophospholipid**	**LysoPC**	568.33966_1.03	LysoPC(22:6)	C30H50NO7P	1.5	1 d	8 d	0.0017
**Glycerophospholipid**	**ether-linked PC**	716.55839_4.77	ePC(O-32:2)	C40H78NO7P	25.3	10 d	1 d	0.0060
**Glycerophospholipid**	**ether-linked PC**	748.62086_5.85	ePC(O-34:0)	C42H86NO7P	1.9	1 d	12 d	<0.0001
**Glycerophospholipid**	**ether-linked PC**	742.57357_4.97	ePC(O-34:3)	C42H80NO7P	2.2	Pre-IR	21 d	0.0268
**Glycerophospholipid**	**ether-linked PC**	792.58893_5.15	ePC(O-36:3)	C44H84NO7P	1.7	12 hr	21 d	<0.0001
**Glycerophospholipid**	**ether-linked PC**	766.57275_5.10	ePC(O-36:5)	C44H80NO7P	1.5	6 d	21 d	0.0008
**Glycerophospholipid**	**ether-linked PC**	796.62085_5.56	ePC(O-38:4)	C46H86NO7P	2.1	3 d	21 d	<0.0001
**Glycerophospholipid**	**ether-linked PC**	794.60319_5.21	ePC(O-38:5)	C46H84NO7P	1.9	12 hr	21 d	<0.0001
**Glycerophospholipid**	**ether-linked PC**	824.64976_5.96	ePC(O-40:4)	C48H90NO7P	2.2	6 d	21 d	<0.0001
**Glycerophospholipid**	**ether-linked PC**	820.61737_5.25	ePC(O-40:6)	C48H86NO7P	2.3	12 hr	21 d	<0.0001
**Glycerophospholipid**	**ether-linked PC**	876.68279_6.18	ePC(O-42:3)	C50H96NO7P	4.3	6 d	21 d	<0.0001
**Glycerophospholipid**	**ether-linked PC**	880.71592_6.53	ePC(O-44:4)	C52H98NO7P	26.4	6 d	12 d	<0.0001
**Glycerophospholipid**	**ether-linked PC**	900.68358_5.89	ePC(O-44:5)	C52H96NO7P	-	6 d	21 d	<0.0001
**Glycerophospholipid**	**ether-linked PC**	756.59019_6.05	ePC(P-35:2)	C43H82NO7P	2.5	12 d	6 d	<0.0001
**Glycerophospholipid**	**ether-linked PC**	818.60229_5.13	ePC(P-40:6)	C48H84NO7P	2.0	12 hr	21 d	<0.0001
**Glycerophospholipid**	**ether-linked PC**	846.63298_5.58	ePC(P-42:6)	C50H88NO7P	4.0	6 d	21 d	<0.0001
**Glycerophospholipid**	**Phosphatidylethanolamine (PE)**	718.53939_5.17	PE(34:0)	C39H78NO8P	2.0	12 hr	12 d	<0.0001
**Glycerophospholipid**	**Phosphatidylethanolamine (PE)**	746.56950_5.58	PE(36:0)	C41H82NO8P	3.0	2 d	4 hr	0.0061
**Glycerophospholipid**	**Phosphatidylethanolamine (PE)**	744.55453_5.23	PE(36:1)	C41H80NO8P	1.3	1 d	6 d	0.0083
**Glycerophospholipid**	**Phosphatidylethanolamine (PE)**	742.53877_4.90	PE(36:2)	C41H78NO8P	1.4	2 d	28 d	0.0019
**Glycerophospholipid**	**Phosphatidylethanolamine (PE)**	740.52232_4.51	PE(36:3)	C41H76NO8P	3.5	8 hr	12 d	0.0014
**Glycerophospholipid**	**Phosphatidylethanolamine (PE)**	738.50670_4.75	PE(36:4)	C41H74NO8P	12.8	Pre-IR	4 hr	0.0183
**Glycerophospholipid**	**Phosphatidylethanolamine (PE)**	764.52460_4.55	PE(38:5)	C43H76NO8P	2.2	12 hr	12 d	<0.0001
**Glycerophospholipid**	**Phosphatidylethanolamine (PE)**	794.57017_5.30	PE(40:4)	C45H82NO8P	1.4	12 hr	12 d	0.0112
**Glycerophospholipid**	**Phosphatidylethanolamine (PE)**	792.55213_4.90	PE(40:5)	C45H80NO8P	1.5	12 hr	12 d	0.0082
**Glycerophospholipid**	**Phosphatidylethanolamine (PE)**	790.53882_4.75	PE(40:6)	C45H78NO8P	1.7	12 hr	21 d	<0.0001
**Glycerophospholipid**	**Phosphatidylethanolamine (PE)**	854.66609_6.09	PE(44:2)	C49H94NO8P	3.7	12 hr	12 d	0.0011
**Glycerophospholipid**	**LysoPE**	452.27718_1.34	LysoPE(16:0)	C21H44NO7P	3.0	1 d	8 d	<0.0001

**Glycerophospholipid**	**LysoPE**	480.30874_1.96	LysoPE(18:0)	C23H48NO7P	2.4	1 d	8 d	<0.0001
**Glycerophospholipid**	**LysoPE**	478.29270_1.46	LysoPE(18:1)	C23H46NO7P	2.9	21 d	28 d	0.0084
**Glycerophospholipid**	**LysoPE**	476.27721_1.13	LysoPE(18:2)	C23H44NO7P	2.4	2 d	12 d	0.0021
**Glycerophospholipid**	**LysoPE**	508.34049_1.87	LysoPE(20:0)	C25H52NO7P	1.5	12 d	4 hr	0.0003
**Glycerophospholipid**	**LysoPE**	506.32445_1.37	LysoPE(20:1)	C25H50NO7P	1.8	12 d	8 hr	<0.0001
**Glycerophospholipid**	**LysoPE**	504.30897_1.09	LysoPE(20:2)	C25H48NO7P	2.7	2 d	8 hr	<0.0001
**Glycerophospholipid**	**LysoPE**	500.27711_1.11	LysoPE(20:4)	C25H44NO7P	2.3	1 d	12 d	<0.0001
**Glycerophospholipid**	**LysoPE**	524.27715_1.06	LysoPE(22:6)	C27H44NO7P	3.5	1 d	Pre-IR	<0.0001
**Glycerophospholipid**	**LysoPE**	564.40516_1.09	LysoPE(24:0)	C29H60NO7P	6.0	2 d	8 hr	<0.0001
**Glycerophospholipid**	**LysoPE**	552.30845_1.01	LysoPE(24:6)	C29H48NO7P	3.5	2 d	12 d	0.0039
**Glycerophospholipid**	**ether-linked PE**	700.52734_5.56	ePE(O-34:2)	C39H76NO7P	2.1	21 d	3 d	0.0175
**Glycerophospholipid**	**ether-linked PE**	698.51183_5.25	ePE(O-34:3)	C39H74NO7P	2.1	10 d	1 d	<0.0001
**Glycerophospholipid**	**ether-linked PE**	730.57299_5.48	ePE(O-36:1)	C41H82NO7P	1.8	Pre-IR	12 d	0.0018
**Glycerophospholipid**	**ether-linked PE**	726.54229_5.10	ePE(O-36:3)	C41H78NO7P	1.6	10 d	21 d	0.0026
**Glycerophospholipid**	**ether-linked PE**	724.52685_5.30	ePE(O-36:4)	C41H76NO7P	2.8	10 d	6 d	<0.0001
**Glycerophospholipid**	**ether-linked PE**	754.57432_6.01	ePE(O-38:3)	C43H82NO7P	4.2	10 d	6 d	<0.0001
**Glycerophospholipid**	**ether-linked PE**	780.58885_5.54	ePE(O-40:4)	C45H84NO7P	3.2	3 d	12 d	<0.0001
**Glycerophospholipid**	**ether-linked PE**	808.61783_5.80	ePE(O-42:4)	C47H88NO7P	9.7	3 d	12 d	<0.0001
**Glycerophospholipid**	**ether-linked PE**	738.50713_4.97	ePE(P-36:4)	C41H74NO8P	2.2	1 d	8 d	0.0187
**Glycerophospholipid**	**ether-linked PE**	766.53909_5.41	ePE(P-38:4)	C43H78NO8P	2.0	1 d	12 d	0.0055
**Glycerophospholipid**	**ether-linked PE**	772.52696_5.13	ePE(P-40:7)	C45H76NO7P	1.6	6 d	21 d	0.0175
**Glycerophospholipid**	**Phosphatidylserine**	816.57515_5.13	PS(38:1)	C44H84NO10P	1.3	12 hr	28 d	0.0009
**Glycerophospholipid**	**Phosphatidylserine**	812.54300_4.62	PS(38:3)	C44H80NO10P	2.9	12 hr	12 d	<0.0001
**Glycerophospholipid**	**Phosphatidylserine**	806.49794_4.99	PS(38:6)	C44H74NO10P	2.8	1 d	8 d	0.0029
**Glycerophospholipid**	**Phosphatidylserine**	846.62575_5.80	PS(40:0)	C46H90NO10P	5.1	Pre-IR	28 d	0.0078
**Glycerophospholipid**	**Phosphatidylserine**	844.60417_5.54	PS(40:1)	C46H88NO10P	2.3	Pre-IR	28 d	0.0322
**Glycerophospholipid**	**Phosphatidylserine**	836.54293_4.51	PS(40:5)	C46H80NO10P	3.1	12 hr	12 d	<0.0001
**Glycerophospholipid**	**Phosphatidylserine**	834.52902_5.41	PS(40:6)	C46H78NO10P	1.9	1 d	8 d	0.0028

**Glycerophospholipid**	**Phosphatidylserine**	830.49455_4.86	PS(40:8)	C46H74NO10P	2.7	1 d	2 d	0.0105
**Glycerophospholipid**	**Phosphatidylserine**	868.60834_5.50	PS(42:3)	C48H88NO10P	3.5	12 hr	28 d	0.0005
**Glycerophospholipid**	**Phosphatidylserine**	866.58854_5.13	PS(42:4)	C48H86NO10P	3.3	12 hr	21 d	<0.0001
**Glycerophospholipid**	**Phosphatidylserine**	864.57471_4.97	PS(42:5)	C48H84NO10P	2.2	8 hr	21 d	<0.0001

Supplementary Table S3. Weight change by kg and % of NHPs.

NHP #	kg (SD -7)	kg (SD 16)	kg (SD 28)	% loss
805356F	3.9	3.9	-	0
805244F	3.8	3.6	3.6	5.3
809095M	4.5	4.5	4.4	2.2
807123M	4.3	4.3	4.0	7.0
805093M	5.3	5.0	-	5.7
804338F	5.3	4.7	4.5	15.1
085115M	4.9	4.8	5.0	0
806428F	4.1	3.9	4.0	2.4

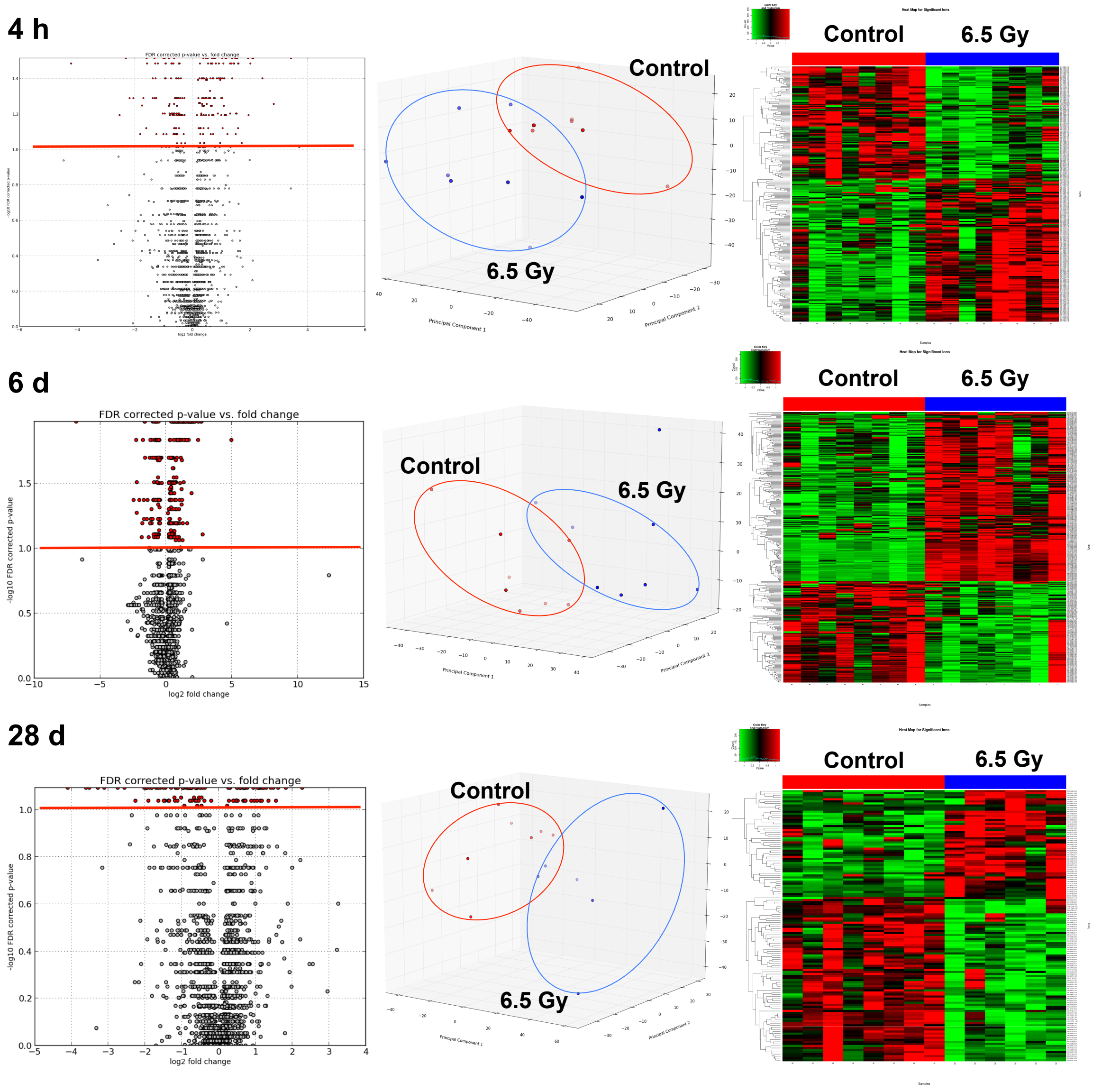

Supplementary Figure S1. Left) Volcano plots; Center) PCA plots; and Right) Heatmaps showing differences between pre-irradiation biosignatures and serum lipid signatures at 4h, 6d, and 28d.

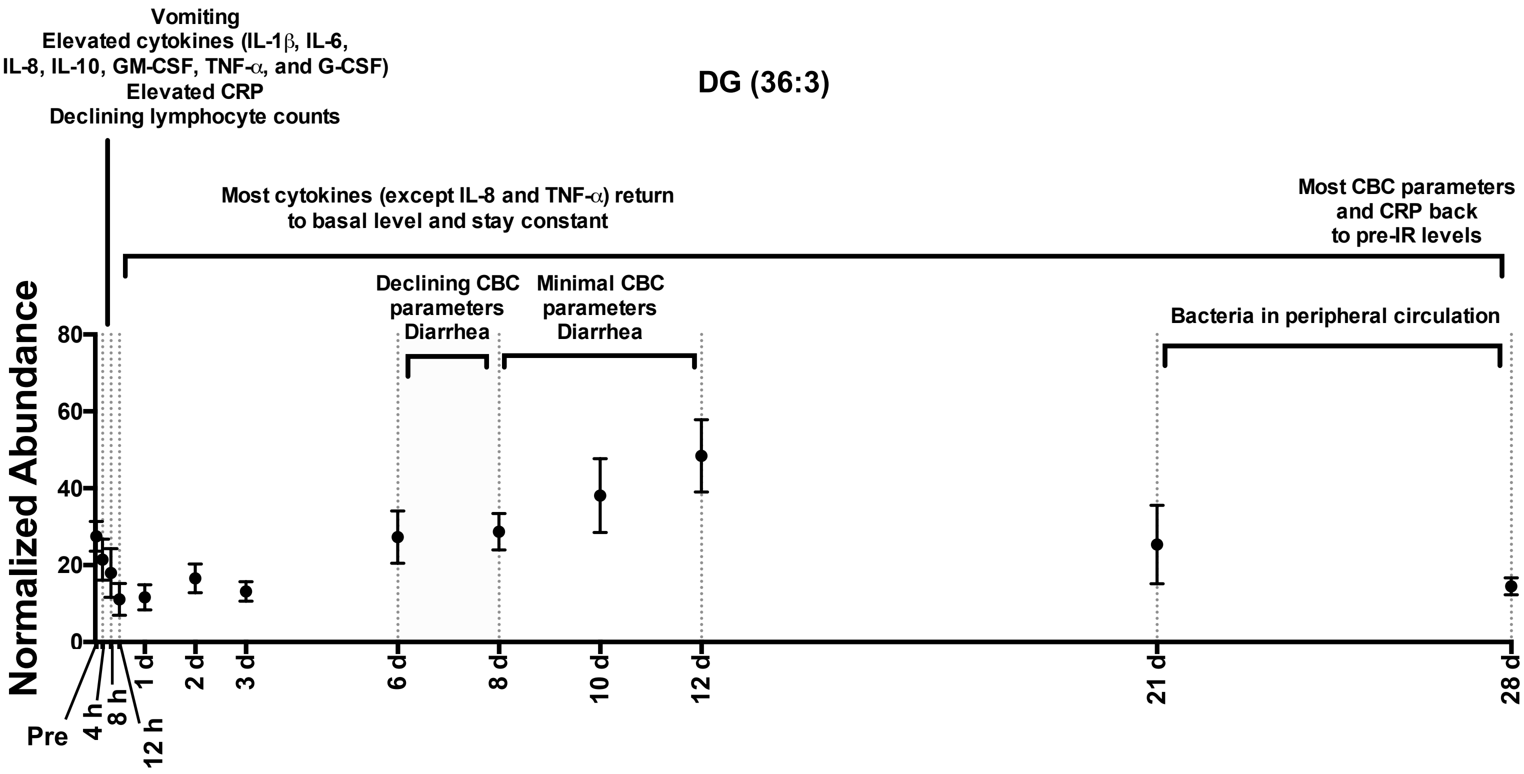

Supplementary Figure S2. Temporal response of DG (36:3) in the serum of NHPs exposed to 6.5 Gy γ-radiation with x-axis corrected for timeline. DGs slightly decrease until ~1 week, increase in concentration during minimal complete blood count (CBC) parameters, and then decrease when bacteria is observed in the peripheral circulation. **Changes in cytokine levels (Supplementary Fig. 8[15]), CBC parameters (Supplementary Fig. 3[15]), and CRP levels (Supplementary Fig. 12[15]) can be found in Singh et al. (2016)[15].**

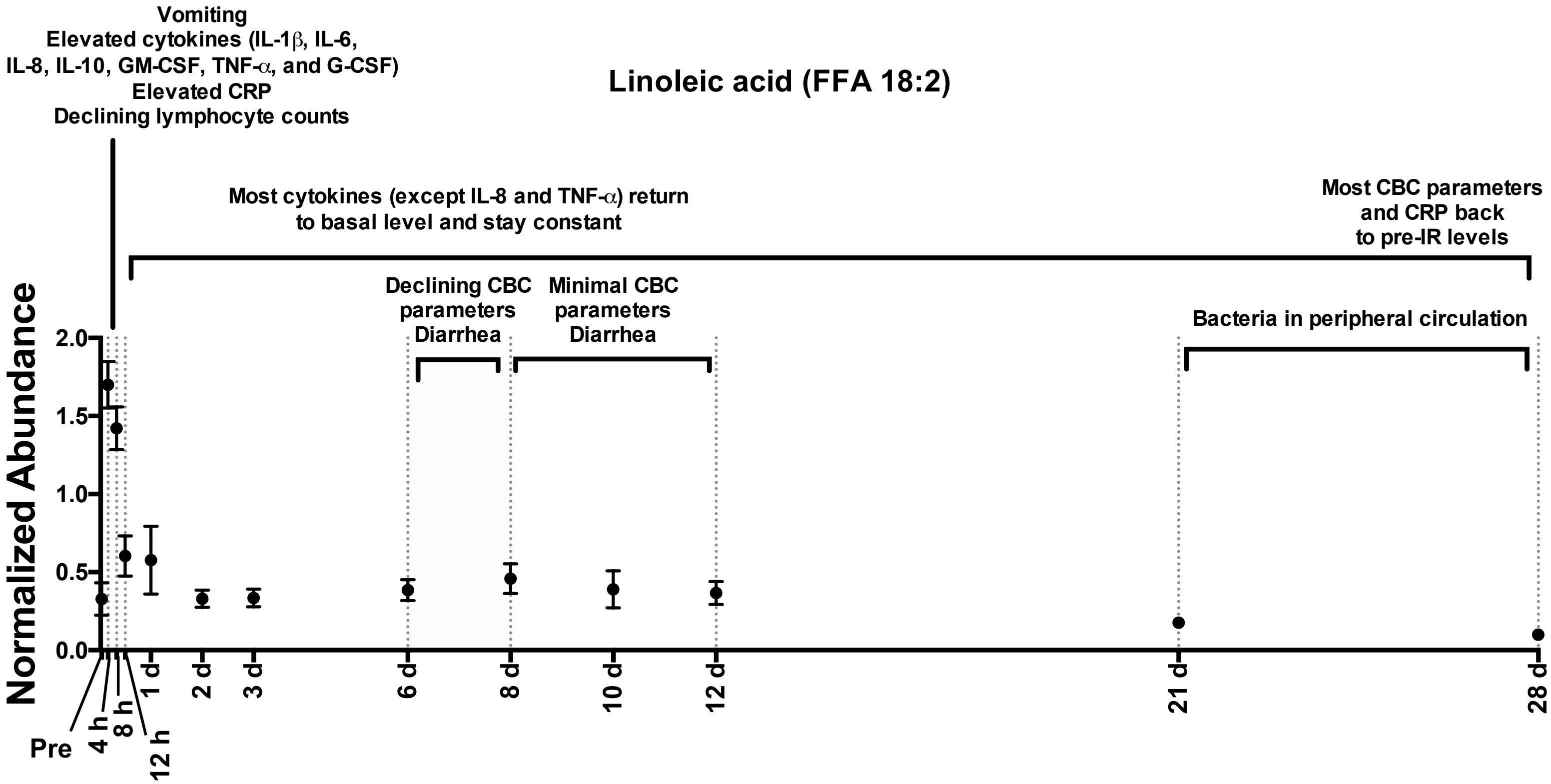

Supplementary Figure S3. Temporal response of FFA (18:2) in the serum of NHPs exposed to 6.5 Gy γ-radiation with x-axis corrected for timeline. FFAs increase during the first 8 h return to pre-irradiation concentrations within 24 h, corresponding to increases in cytokine levels. The lowest levels are observed when bacteria is present in the peripheral circulation. **Changes in cytokine levels (Supplementary Fig. 8[15]), CBC parameters (Supplementary Fig. 3[15]), and CRP levels (Supplementary Fig. 12[15]) can be found in Singh et al. (2016)[15].**
